# Supplementary material for: Multimodal imaging reveals a lysosomal drug reservoir that drives heterogeneous distribution of PARP inhibitors
Source: Nat Commun. 2026 Mar 17;17:4086. doi: 10.1038/s41467-026-70558-1 (PMC13144616; doi:10.1038/s41467-026-70558-1)
Supplement: Supplementary file 1 — Supplementary Information [file 41467_2026_70558_MOESM1_ESM.pdf]

Supplementary Information

- Supplementary Table 1: Non-commercial buffers and solutions
- Supplementary Table 2: Immunohistochemistry primary antibodies
- Supplementary Table 3: Spatial Transcriptomics staining reagents
- Supplementary Table 4: Drug stock details
- Supplementary Table 5: Immunocytochemistry primary antibodies
- Supplementary Table 6: Immunocytochemistry secondary antibodies
- Supplementary Table 7: Operetta CLS High Content Analysis System channels
- Supplementary Table 8: Expression plasmids and details for viral vector production

- Supplementary Figure 1: Optimisation of MSI platforms for drug quantification in PDE sections dosed ex-vivo (associated with main Figure 1)
- Supplementary Figure 2: Multimodal molecular imaging of serial PDE sections (associated with main Figure 1)
- Supplementary Figure 3: High- and low-drug spatial sampling in PDEs for genomic analysis (associated with main Figure 2)
- Supplementary Figure 4: Transcriptomic profiling identifies lysosomal gene enrichment associated with niraparib and rucaparib accumulation in PDEs (associated with main Figure 2)
- Supplementary Figure 5: Differential sensitivity and single-cell response heterogeneity to PARP inhibitors across ovarian cancer cell lines (associated with main Figure 3)
- Supplementary Figure 6: Spatiotemporal kinetics and heritability of rucaparib accumulation in PEO1 cells (associated with main Figure 3)
- Supplementary Figure 7: Lysosomal content determines intracellular rucaparib levels (associated with main Figure 4)
- Supplementary Figure 8: Palbociclib mechanism of action and the relationship between nuclear rucaparib levels and DNA damage (associated with main Figure 5)
- Supplementary Figure 9: Modulation of rucaparib nuclear bioavailability via pharmacological inhibition of lysosomal function (associated with main Figure 6)
- Supplementary Figure 10: Impact of lysosomal accumulation on intracellular PARP inhibitor concentrations (associated with main Figure 6)

Supplementary Table 1: Non-commercial buffers and solutions

Name	Constituents
0.4% SRB	0.4% sulforhodamine B (SRB) in 1% acetic acid
Blocking and permeabilization solution	2% bovine serum albumin (BSA) 0.5% Triton-X in PBS
Buffer B	75% acetonitrile, 20% water, 5% DMSO with + 0.1% ferulic acid (FA)
DHB matrix	20 mg/mL 2,5-dihydroxybenzoic acid (DHB) in 50% acetone with 0.1% trifluoroacetic acid (TFA)
Laemmli buffer	1M Tris, 10% SDS, 10% Glycerol, 5% β-mercaptoethanol, 0.0002% bromophenol blue
NBF stop buffer	101 mM Tris, 100 mM Glycine in DEPC treated water
PBS	137 mM NaCl, 2.7 mM KCl, 8 mM Na2HPO4, 1.5 mM KH2PO4
Stringent wash	50% deionized formamide 2X SSC buffer
TBS	50 mM Tris, 150 mM NaCl
Tris-glycine running buffer	2.5 mM Trizma Base, 0.2 M glycine, 0.01% SDS

Supplementary Table 2: Immunohistochemistry primary antibodies

**Target**	**Antibody Clone**	**Species**	**Dilution**
Wilms Tumour Protein (WT1)	Abcam, ab89901 CANR9(IHC)-56-2	Rabbit	1:500
Pax8	Abcam, ab189249 EPR13511	Rabbit	1:1000
Phospho histone H2A.X (Ser139)	CST, 2577	Rabbit	1:400
Cleaved Caspase-3 (Asp175)	CST, 9664 5A1E	Rabbit	1:100

Supplementary Table 3: Spatial Transcriptomics staining reagents

Target	Antibody	Clone	Fluorophore (nm)	Concentration
Nucleic acid (SYTO-13)	GeoMx Solid Tumor TME Morphology Kit	n/a	488	1:10 dilution
Pan cytokeratin (PanCK)	Novus, NBP2-33200	AE1+AE3	532	80 µg/ml
CD45	Novus, NBP2-34528	2B11+PD7/26	594	320 µg/ml
Alpha-smooth muscle actin (α-SMA)	Abcam, ab267537	SP171	647	0.5 µg/ml

Supplementary Table 4: Drug stock details

Compound	Catalogue number	Stock concentration	Vehicle
Bafilomycin A1	Sigma 5.08409	0.1mg/mL	DMSO
Chloroquine	Sigma C6628	50 mg/mL	PBS
EN6	MCE HY-128892	50 mM	DMSO
Niraparib	Selleck S2741	100 mM	DMSO
Olaparib	Selleck S1060	100 mM	DMSO
Palbociclib	ApexBio A8316-APE	1 mM	DMSO
Puromycin	Merck 540222	1mg/mL	H2O
Rucaparib	ApexBio A4156-APE	100 mM	DMSO

Supplementary Table 5: Immunocytochemistry primary antibodies

Target	Antibody Clone	Species	Dilution
Phospho histone H2A.X (Ser139)	CST 2577	Rabbit	1:1000
α-Tubulin Sigma	T9026 DM1A	Mouse	1:1000

Supplementary Table 6: Immunocytochemistry secondary antibodies

Antibody	Species	Invitrogen catalogue number
Alexa Fluor 488	Rabbit	A21206
Alexa Fluor 555	Rabbit	A31572
Alexa Fluor 568	Mouse	A10037
Alexa Fluor 647	Mouse	A31571

Supplementary Table 7: Operetta CLS High Content Analysis System channels

Channel	Excitation wavelength range (nm)	Emission wavelength range (nm)
Alexa Fluor 488	460-490	500-550
Alexa Fluor 555	530-560	510-650
Alexa Fluor 568	530-560	510-650
Alexa Fluor 647	615-645	655-760
Hoechst	360-400	410-480
eGFP	460-490	500-550

Supplementary Table 8: Expression plasmids and details for viral vector production

**Name**	**Backbone**	**Vector type**	**Bacterial resistance**	**Addgene reference**	**Gene insert**
**pLJM1-EGFP**	pLJM1	Mammalian expression (3rd generation lentiviral)	Ampicillin 100 µg/mL	#19319	eGFP
**pEGFP-N1-TFEB**	pEGFP-N1	Mammalian expression (transient)	Kanamycin 100 µg/mL	#38119	TFEB-eGFP

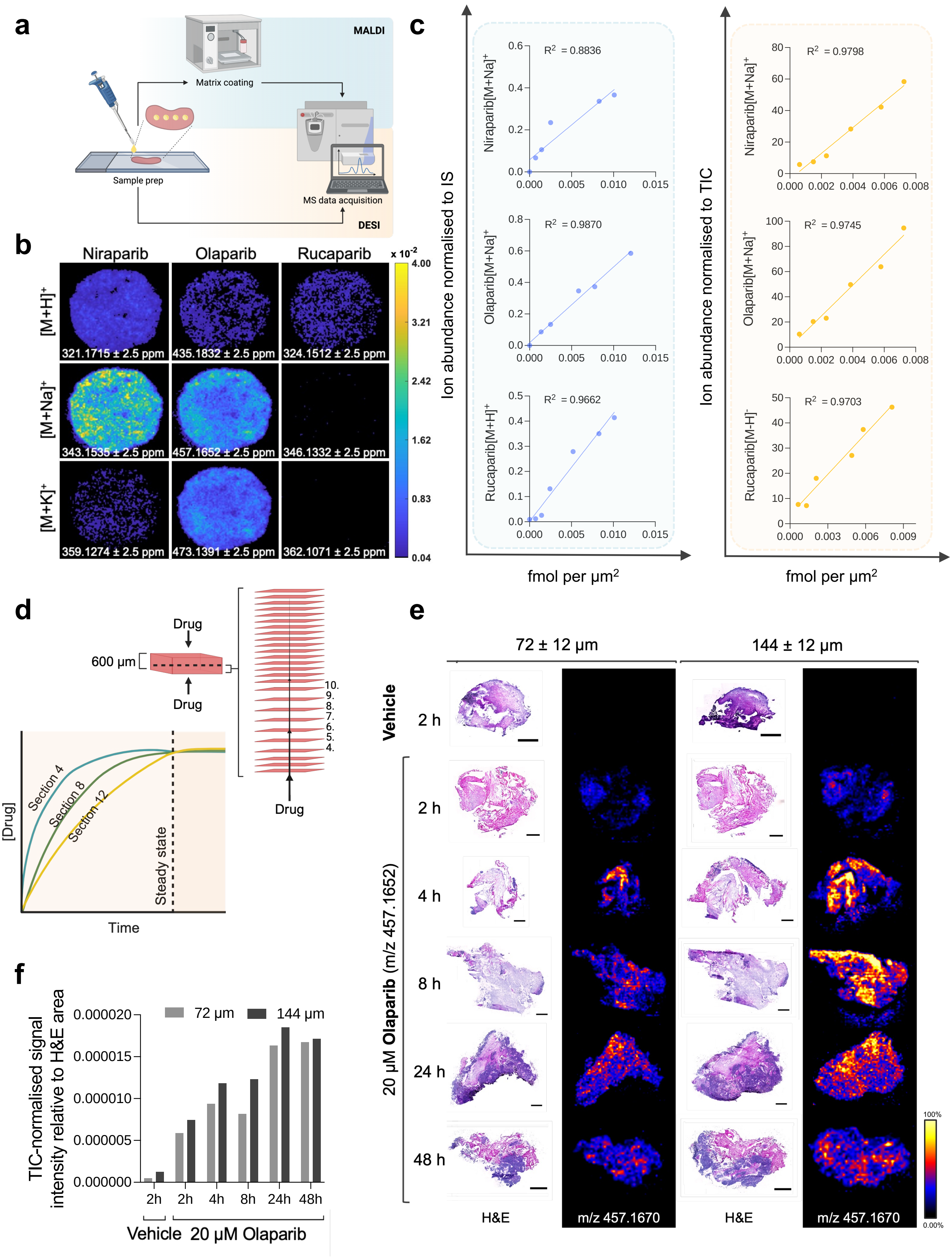

Supplementary Figure 1

Supplementary Figure 1

Optimisation of MSI platforms for drug quantification in PDE sections dosed ex-vivo (associated with main Figure 1)

A) Schematic representation of mass spectrometry imaging calibration line generation process in Atmospheric-Pressure Matrix-Assisted Laser-Desorption ionisation (AP-MALDI) and Desorption Electrospray Ionisation (DESI) platforms. Calibrants were spotted on top of un-dosed HGSOC tissue sections. Created in BioRender. Ramirez moncayo, C. (2026) https://BioRender.com/7m74wgk.

B) Adducts of niraparib, olaparib and rucaparib detected in AP-MALDI platform, in positive ion mode with 2,5-dihydroxybenzoic acid (DHB) matrix. Total Ion Chromatogram (TIC)-normalisation and a tolerance window of 2.5 parts per million (ppm) was applied.

C) AP-MALDI (left) and DESI (right) calibration lines generated in positive or negative (DESI, rucaparib only) ion modes. Data were normalised to drug-matched internal standards (IS) and TIC for each respective modality.

D) Patient-Derived Explant (PDE) dosing timepoint optimisation schematic. Tissue explants were dosed with olaparib at 20 µM for up to 48 hours to determine time required for the compound to homogeneously diffuse through the top 150 µm of tissue. This depth was selected to match the number of 10 µm sections typically used in downstream analyses, ensuring that drug exposure reached steady state across all sections. Created in BioRender. Ramirez moncayo, C. (2026) https://BioRender.com/glxwlvm.

E) For each sample, the 6th and 12th section (72 and 144 µm deep respectively) were thaw-mounted onto a glass slide for spectral data acquisition using DESI in positive ion mode. Comparison between tissue architecture as seen through haematoxylin and eosin staining (H&E, scale bar = 750 µm) and olaparib signal throughout time is shown ($[M+Na]^+$ adduct, experimental m/z 457.1670, mass accuracy = -3.96 ppm).

F) Quantification of olaparib in 6th and 12th sections throughout time, calculated as the average TIC-normalised ion intensity value per tissue section area, thresholded through haematoxylin and eosin staining optical density.

For B and E, colour scales depict relative drug intensity.

All optimisation experiments were performed on tissue slices from 1 patient.

Source data are provided as a Source Data file.

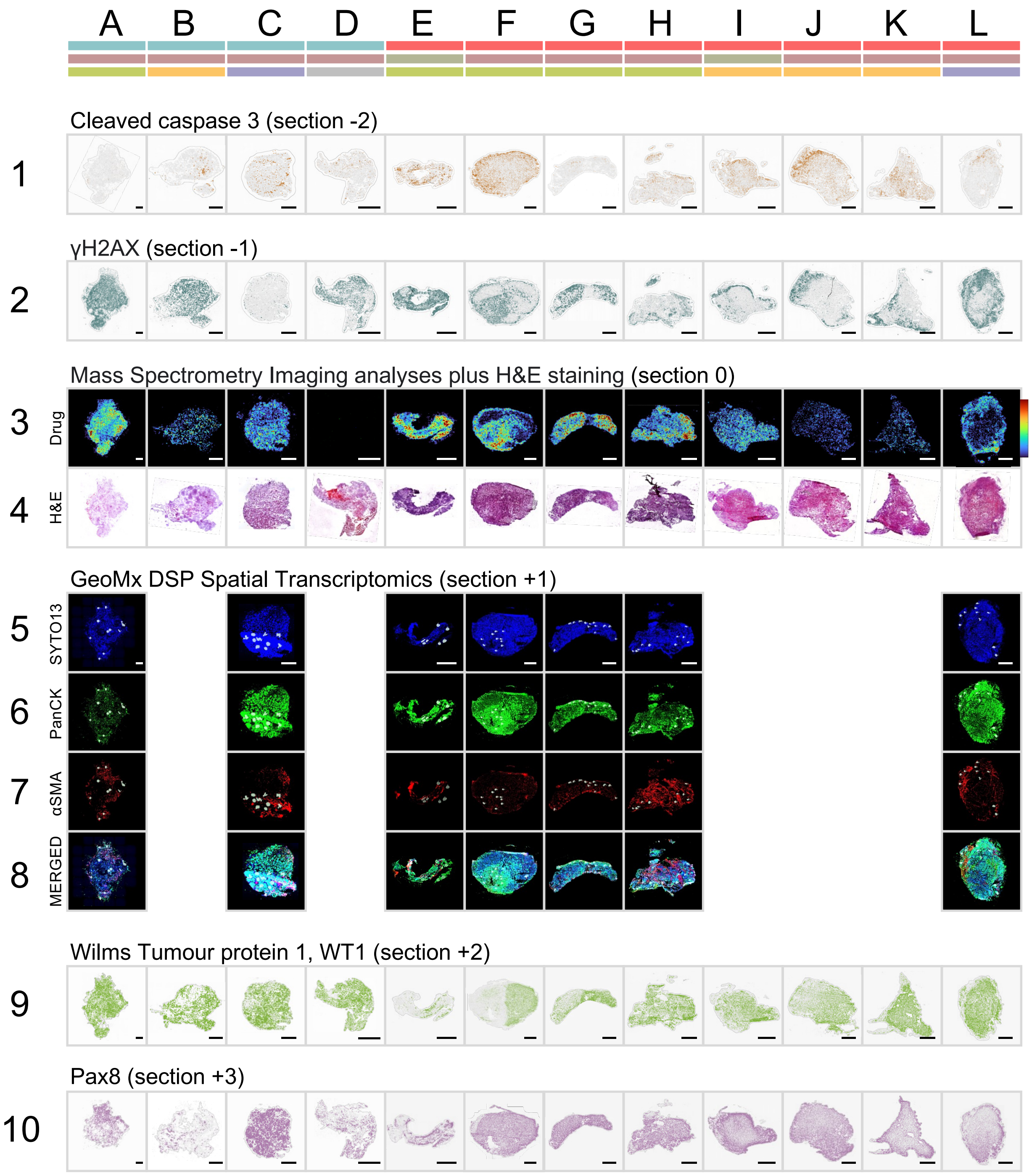

Supplementary Figure 2

Multimodal molecular imaging of serial PDE sections (associated with main Figure 1)

2-part figure depicting serial sections from all treated Patient-Derived Explants (PDEs) used to perform drug MSI signal analysis, haematoxylin and eosin (H&E) staining, immunoistochemical (IHC) analyses, and where relevant, GeoMx spatial transcriptomics, where chosen ROIs are shown. Key indicating colour codes for patient, tumour site and drug in part 2 of figure. For IHC analyses (rows 1, 2, 9 and 10) pseudo-colour overlays highlight positive cells compared to negative controls (no primary antibody). For MSI data, colour scales depict relative drug intensity. Source data for MSI are provided as a Source Data file.

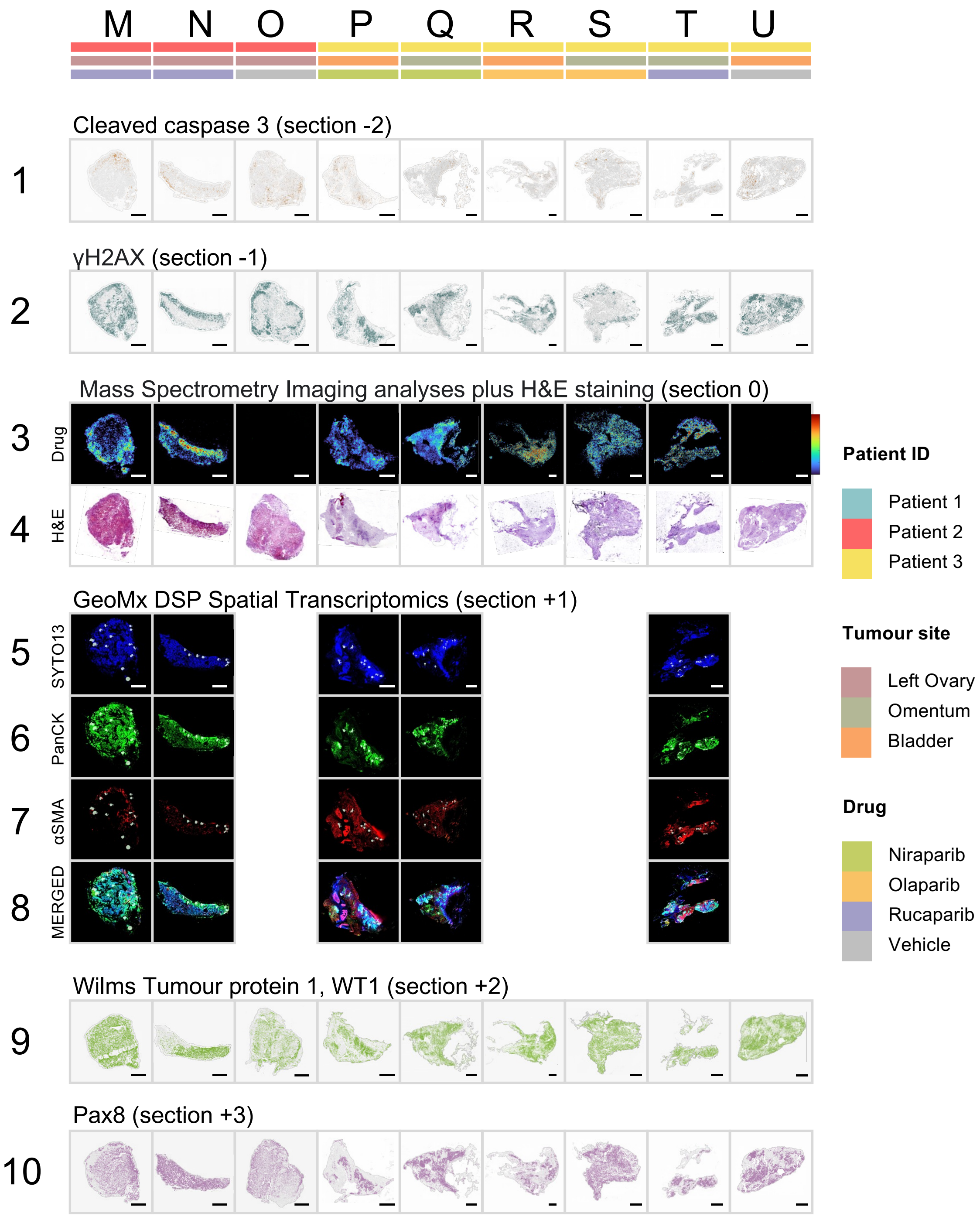

Supplementary Figure 2 continued

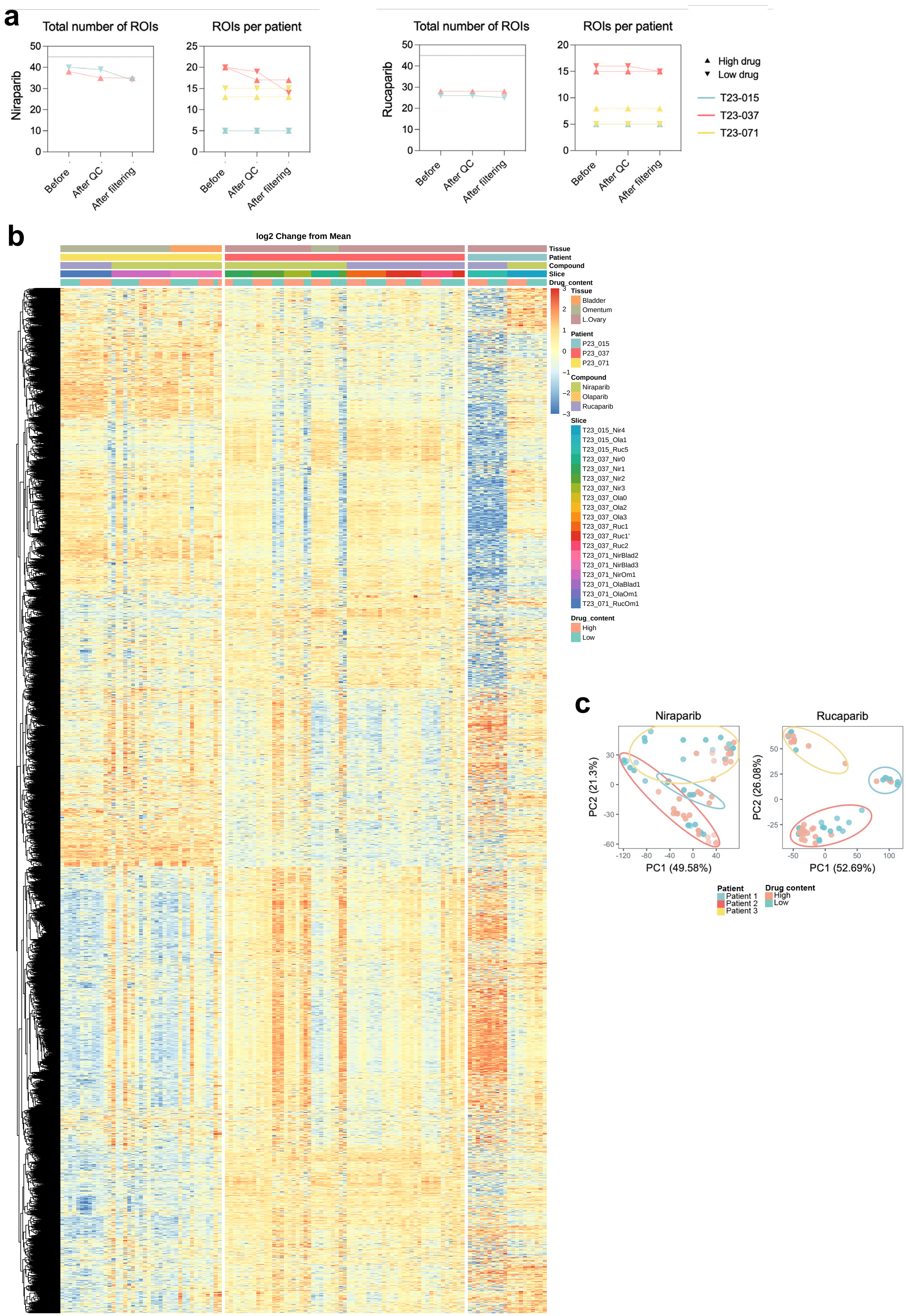

Supplementary Figure 3

Supplementary Figure 3

High- and low-drug spatial sampling in PDEs for genomic analysis (associated with main Figure 2)

A) Regions of interest (ROI) included in GeoMx spatial transcriptomics study. On average, 5 'high-' and 5 'low-drug' ROIs were chosen across Patient-Derived Explant (PDE) samples from three patients, resulting in approximately 45 ROIs per compound and drug content. Per compound, the total number of ROIs obtained is shown on the left, with a breakdown by patient displayed on the right, before and after data quality control (QC) and filtering. QC involved applying a threshold of 100,000 raw reads and 50% sequencing saturation, discarding any ROI that fell below these criteria. ROIs with less than 5% of targets above the limit of quantification were filtered out.

B) Supervised hierarchical clustering of GeoMx spatial transcriptomics dataset. Samples were grouped by Patient ID before hierarchical clustering analysis performed using Euclidean distance. All 11224 targets are shown.

C) Principal component analysis of niraparib and rucaparib datasets. ROIs are coloured based on drug content, with outlines representing patient clusters.

Source data are provided as a Source Data file.

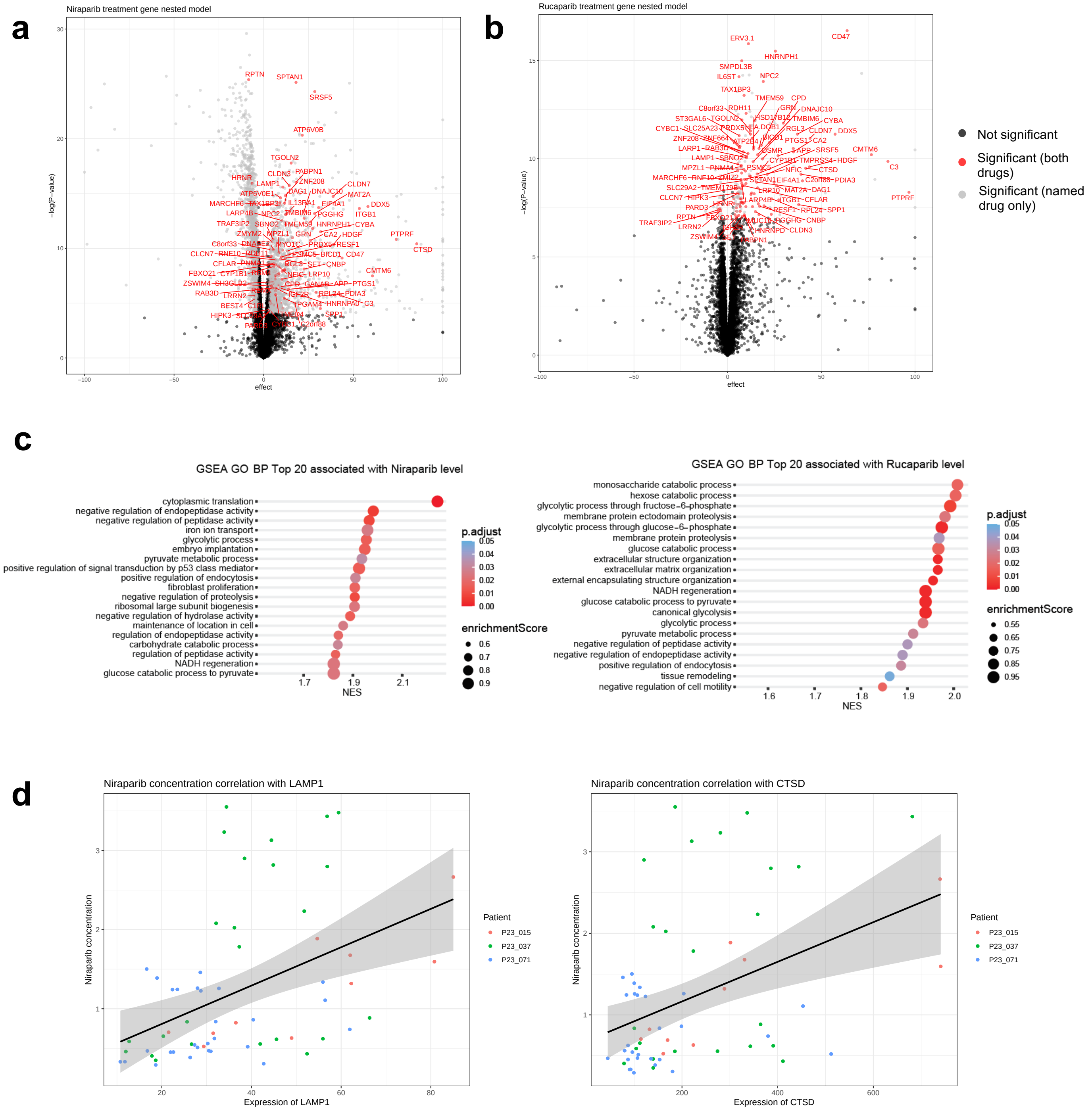

Gene	Pearson rho	*P* Value
LAMP1	0.442	3.2e-4
CTSD	0.421	6.5e-4

Supplementary Figure 4

Supplementary Figure 4

Transcriptomic profiling identifies lysosomal gene enrichment associated with niraparib and rucaparib accumulation in PDEs (associated with main Figure 2)

A) Volcano plot depicting genes identified in niraparib linear mixed model regression analysis. Genes significantly associated with increasing drug illustrated in grey points ($p < 0.05$) , genes illustrated with red points (and text) are also significant in the rucaparib analysis.

B) Volcano plot depicting genes identified in rucaparib linear mixed model regression analysis. Genes significantly associates with increasing drug illustrated in grey points ($p < 0.05$), genes illustrated with red points (and text) are also significant in the niraparib analysis.

C) Gene set enrichment analysis (GO biological processes) for genes significantly associated with increasing levels of niraparib (left) and rucaparib (right) within PDE samples in linear mixed model regression analysis.

D) Scatter plots depicting expression of lysosomal genes relative to niraparib concentration from PDE experiments, each dot represents an ROI and patients are colour-coded. The grey shaded areas represent the standard error SE of the linear fit. Pearson correlation coefficients are indicated for each plot.

N-values displayed in Supplementary Fig. 3a. Source data are provided as a Source Data file.

a

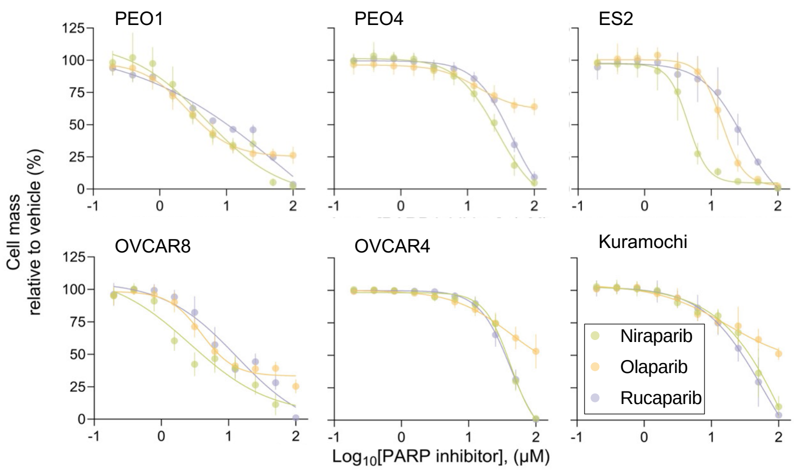

b

IC_{50} (μM)	Niraparib	Olaparib	Rucaparib
PEO1	6.5	10	8.5
PEO4	38	15	40
ES2	4.5	14.5	30
OVCAR8	2.5	4	12.5
OVCAR4	42	38.5	42
Kuramochi	34	75	26

c

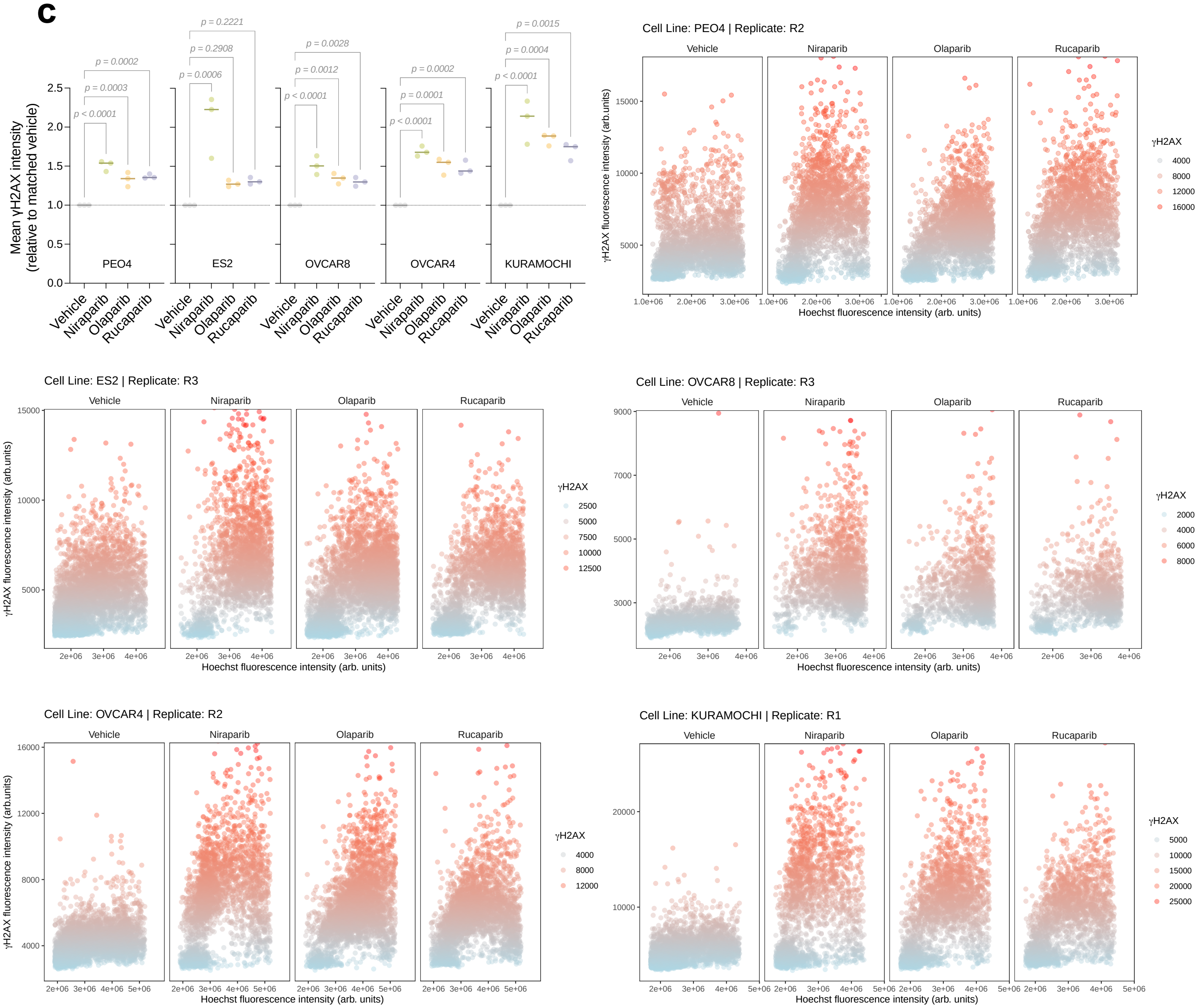

Supplementary Figure 5

Supplementary Figure 5

Differential sensitivity and single-cell response heterogeneity to PARP inhibitors across ovarian cancer cell lines (associated with main Figure 3)

A) Dose response curves to all three PARP inhibitors (niraparib, olaparib and rucaparib) for 6 OvCa cell lines, measured by SRB assays. Data depicts biological means ± SD (n = 3).

B) IC_{50} values of OvCa cell lines measured by SRB assays. Estimated values by fitting appropriate non-linear regression model in Graphpad Prism: [Inhibitor] vs. response – Variable slope (four parameters) for all lines except Kuramochi, which was fitted with the [Inhibitor] vs. normalized response model.

C) Heterogeneity in response to PARP inhibitors measured by γH2AX level in OvCa cell lines. Each cell line was dosed at their corresponding IC_{50} values for each PARP inhibitor for 24h. Biological γH2AX means (n = 3) relative to the mean of the vehicle control within each replicate are depicted per cell line, followed by representative single-cell scatter plots of γH2AX and Hoechst fluorescence intensities. P-values were determined by a two-sided, repeated-measures one-way ANOVA with Dunnett's multiple comparison test.

Source data are provided as a Source Data file.

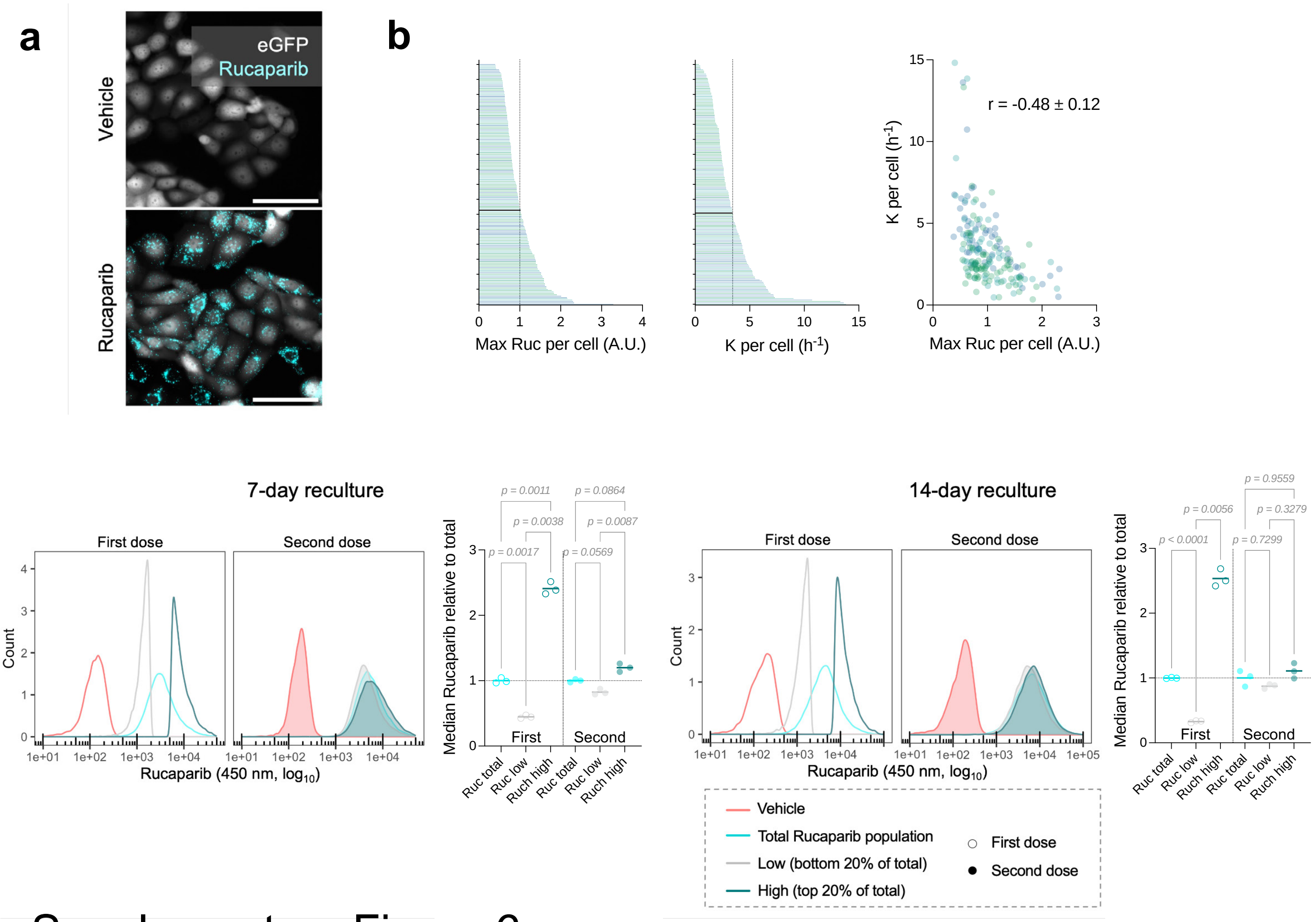

Supplementary Figure 6

Spatiotemporal kinetics and heritability of rucaparib accumulation in PEO1 cells (associated with main Figure 3)

A) eGFP-PEO1 treated with vehicle-only or rucaparib at IC_{50} concentration for 24h, 100 μm scale bar.

B) Right; live PEO1 single-cell rucaparib accumulation kinetic parameters, estimated with a one-phase association non-linear model, fitted over mean rucaparib fluorescence intensity per cell over time. Black line marks the average values. Left; Pearson's correlation coefficient between rate constant K and maximum rucaparib per cell was calculated over 3 colour-coded independent biological replicates.

C) Ruc_{High} and Ruc_{Low} PEO1s (top and bottom 20% of the rucaparib-treated population respectively) were FACS-sorted in triplicates with total rucaparib and vehicle-only controls after a 2h treatment. Cells were re-cultured in drug-free medium for 7 and 14 days and then re-dosed to analyse rucaparib signal with respect to its original distribution (left). Relative drug levels for each population are shown on the right. 'First' denotes initial rucaparib signal. 'Second' refers to re-dosed levels after re-culture. Significance by two-sided Welch one-way ANOVA, followed by Dunnett's.

Source data are provided as a Source Data file.

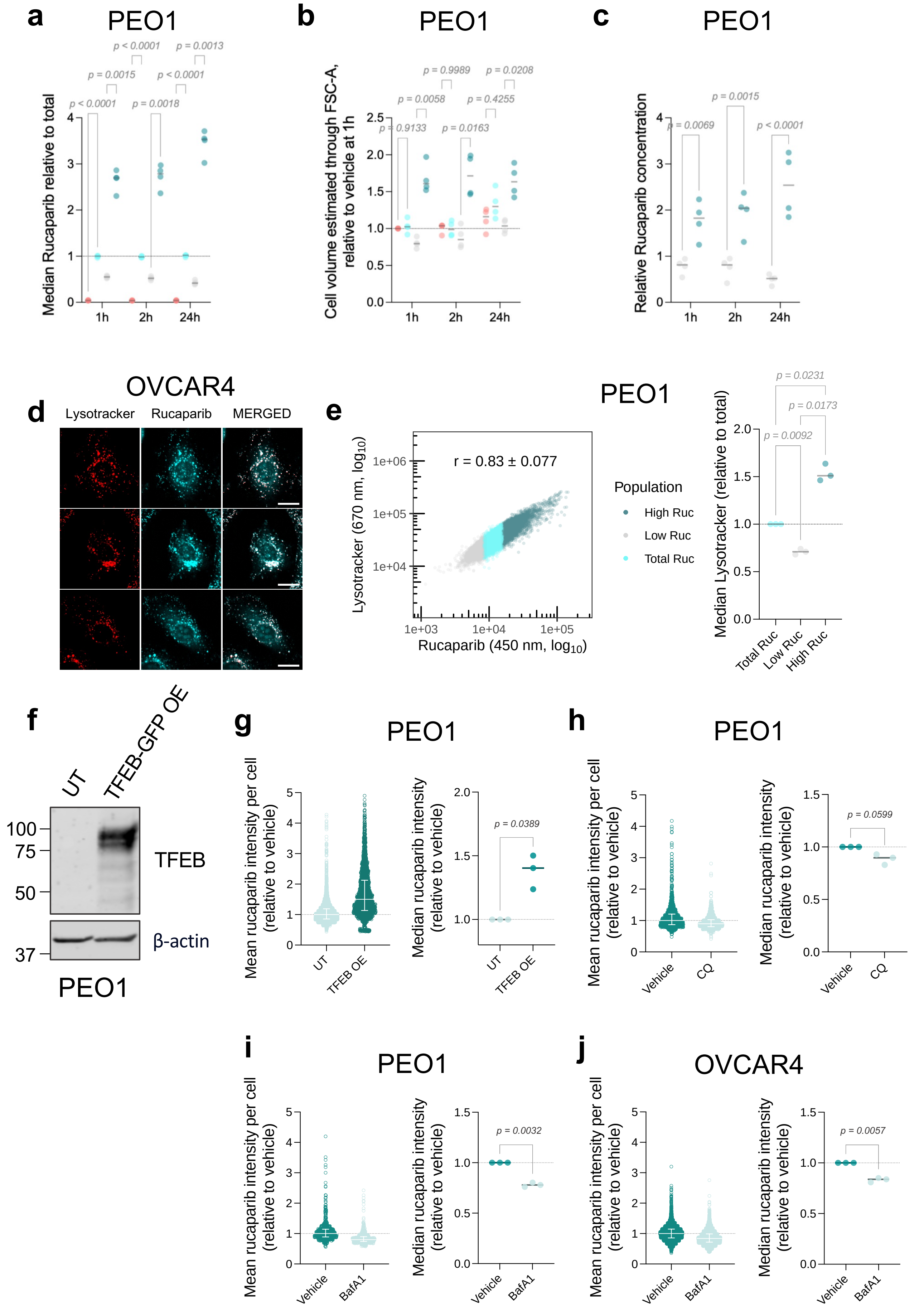

Supplementary Figure 7

Supplementary Figure 7

Lysosomal content determines intracellular rucaparib levels (associated with main Figure 4)

A) Median drug signal intensity per FACS gate in PEO1 cells treated and sorted based on rucaparib fluorescence intensity. Rucaparib signal was normalised to the signal intensity of the rucaparib-treated, ungated population (Ruc total) per time point and biological replicate (n = 4).

B) PEO1 cell volume per FACS gate. Cell volume was estimated through median FSC-A per gate, which is proportional to cell diameter. Volumes were normalised, per biological replicate (n = 4), to that of 1h vehicle-treated cells.

C) Rucaparib concentration per FACS gate of PEO1 cells. Rucaparib signal intensity was normalised to cell volume, estimated through FSC-A as a proxy of cell diameter (n = 4).

D) Example of colocalisation of rucaparib with the lysosome at 60min post dosing in OVCAR4 cells. Scale bar = 20 µm.

E) FACS-based correlation between lysosomal and rucaparib contents per PEO1 cell, after 1.5h dosing of both compounds (left). Cells were gated as before (see Fig. 4a), and median LysoTracker signal per gate was plotted relative to each biological replicate's total signal.

F) Western blot to demonstrate TFEB-GFP overexpression in PEO1 cells.

G) Left; rucaparib signal per cell in untransfected PEO1 cells or cells transfected with TFEB-GFP. Right; median rucaparib levels per biological replicate.

H) Left; rucaparib signal per PEO1 cell following CQ treatment. Right; median rucaparib levels.

I) Left; rucaparib signal per PEO1 cell following BafA1 treatment. Right; median rucaparib levels.

J) Left; rucaparib signal per OVCAR4 cell following BafA1 treatment. Right; median rucaparib levels.

For A-C and E, two-way ANOVA (or one-way for E) with the Geisser-Greenhouse correction and Tukey's (A, B, E) or Sidak (C) multiple comparison test were applied to evaluate statistical significance. For G–J, Welch two-sample t-test was applied to assess significance, and left panels show median ± interquartile range of a representative biological replicate. Unless otherwise stated, all experiments were performed in biological triplicates. Source data are provided as a Source Data file.

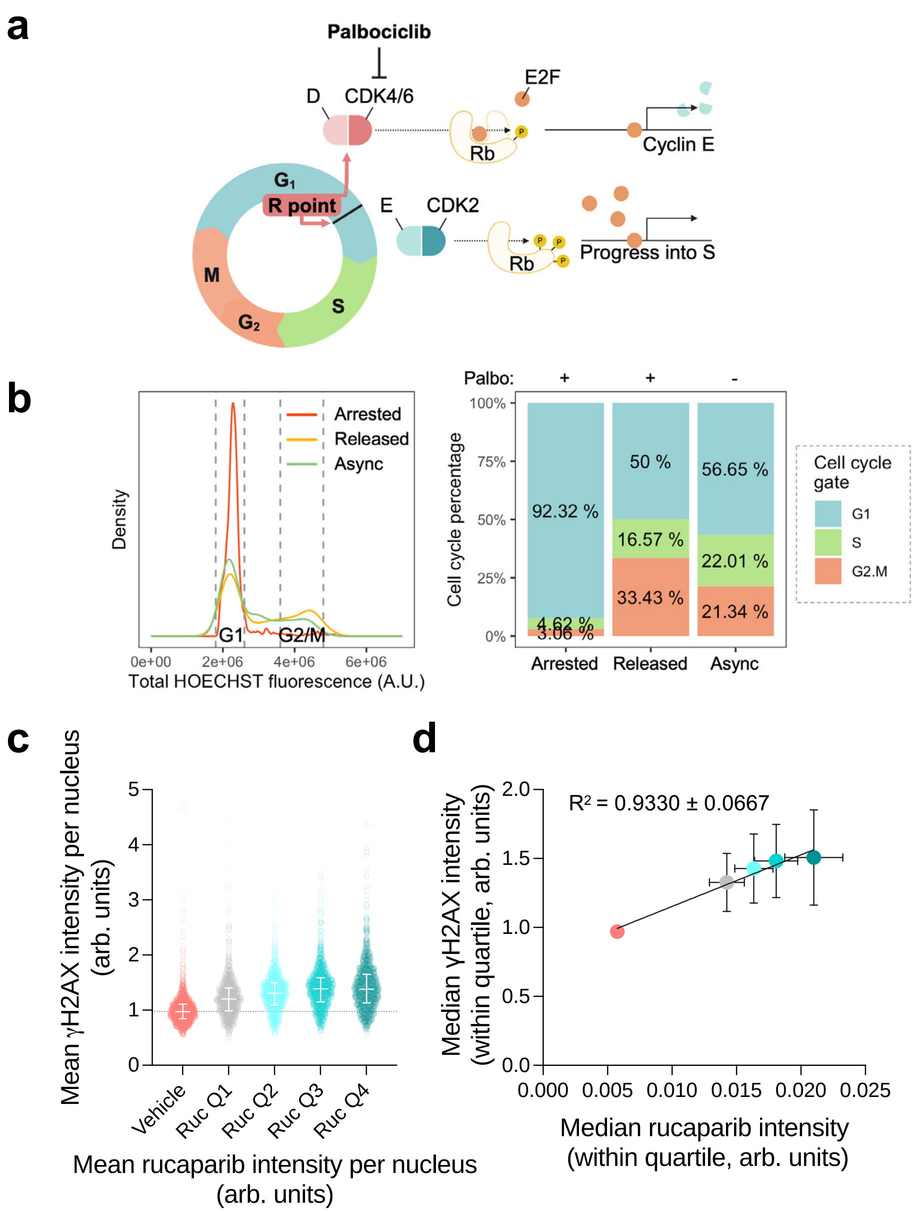

Supplementary Figure 8

Palbociclib mechanism of action and the relationship between nuclear rucaparib levels and DNA damage (associated with main Figure 5)

A) Schematic representation of palbociclib mechanism of action. Created in BioRender. Ramirez moncayo, C. (2026) https://BioRender.com/ejp6dm9.

B) PEO1 cells were treated with palbociclib or vehicle-only for 24h and cultured in drug-free medium for an additional 24h before fixation and DNA staining with hoechst. Cell cycle gating was performed based on hoechst sum fluorescence intensity to estimate percentage of cells in each cell-cycle phase (n = 3).

C) OVCAR4 cell population was subdivided into quartiles based on nuclear rucaparib signal to compare γH2AX levels between groups (median ± interquartile range). Data representative of n = 3 biological replicates.

D) In OVCAR4 cells, median γH2AX is linearly related to median intracellular rucaparib within the quartile. Mean ± SD from n = 3 biological replicates are plotted.

Source data are provided as a Source Data file.

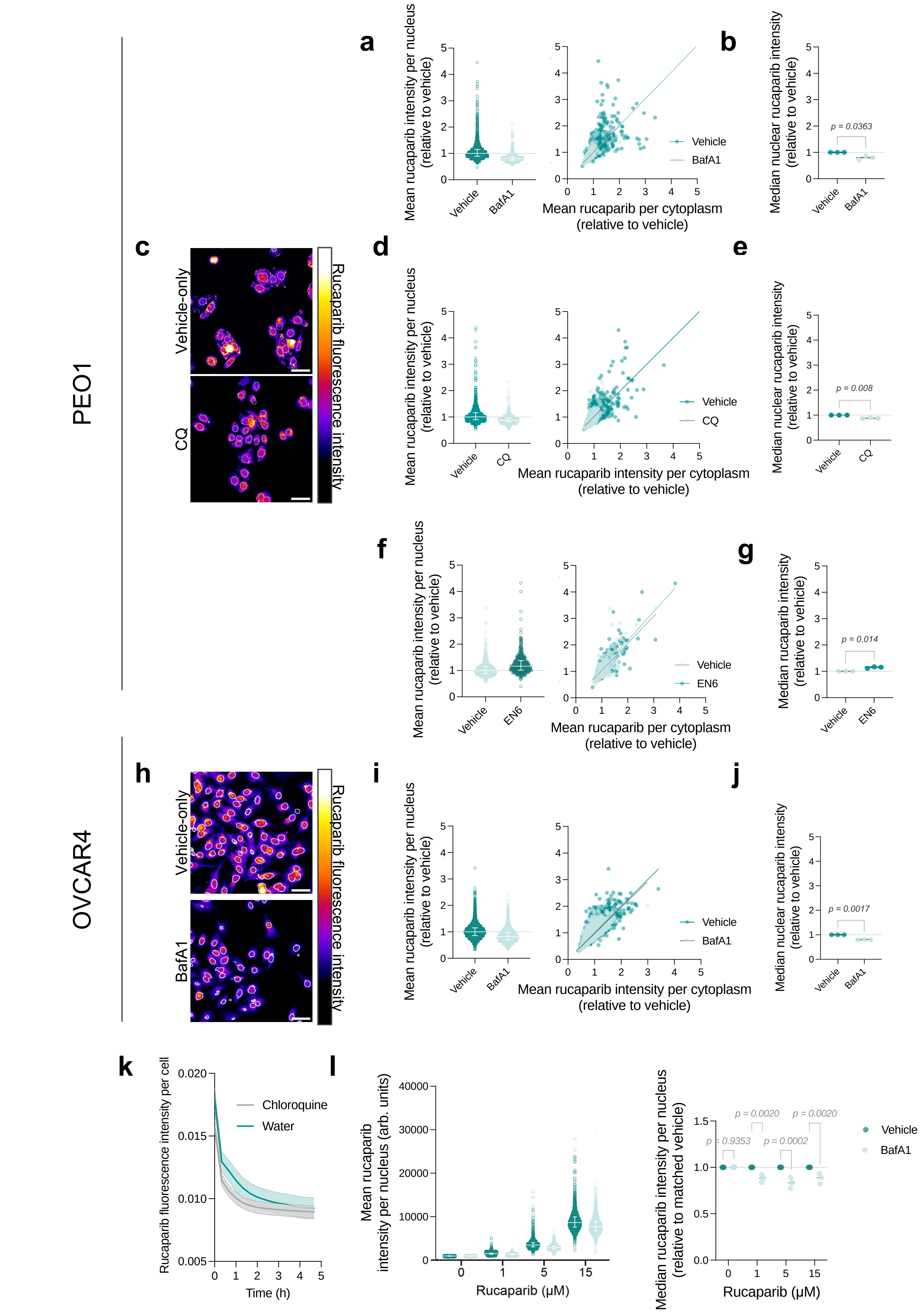

Supplementary Figure 9

Supplementary Figure 9

Modulation of rucaparib nuclear bioavailability via pharmacological inhibition of lysosomal function (associated with main Figure 6)

A) Left; quantification of nuclear levels of rucaparib (per PEO1 cell), ± bafilomycin (BafA1) pre-treatment. Right; correlation between nuclear and cytoplasmic drug ($p < 0.05$).

B) Median nuclear rucaparib per PEO1 biological replicate pre-treated with ± BafA1.

C) Representative images of rucaparib in PEO1 cells after 1h ± chloroquine (CQ) pre-treatment. Dotted white lines outline the nucleus. Scale bar represents 50 μm.

D) Left; quantification of nuclear levels of rucaparib (per PEO1 cell), ± EN6. Right; correlation between nuclear and cytoplasmic drug.

E) Median nuclear rucaparib per PEO1 biological replicate pre-treated with ± EN6.

F) Left; quantification of nuclear levels of rucaparib (per PEO1 cell), ± CQ pre-treatment. Right; correlation between nuclear and cytoplasmic drug.

G) Median nuclear rucaparib per PEO1 biological replicate pre-treated with ± CQ.

H) Representative images of rucaparib in OVCAR4 cells after 1h with or without pre-treatment with BafA1. Dotted white lines outline the nucleus. Scale bar represents 50 μm.

I) Left; quantification of nuclear levels of rucaparib (per OVCAR4 cell), ± BafA1 pre-treatment. Right; correlation between nuclear and cytoplasmic drug.

J) Median nuclear rucaparib per OVCAR4 biological replicate pre-treated with ± BafA1.

K) Rucaparib fluorescence loss after washout in PEO1 cells pre-treated with ± CQ. Data show mean fluorescence intensity per cell ± SD ($n = 6$ replicate wells) and is representative of 2 biological replicates.

L) PEO1 cells were pre-treated with ± BafA1 (1h) followed by rucaparib (0, 1, 5, or 15 μM) for 1h. Left; nuclear rucaparib per cell. Right; median rucaparib per nucleus per biological replicate, normalised to matched vehicle controls. 2-way ANOVA and Sidak multiple comparison test were applied to evaluate statistical significance.

All data was collected across $n = 3$ biological replicates. For A, D, F, I, L (left), data depict median ± interquartile range and are a representative replicate. For B, E, G, J, significance was assessed by Welch two-sample t-test. For C, H, colour scales depict drug fluorescence intensity.

Source data are provided as a Source Data file.

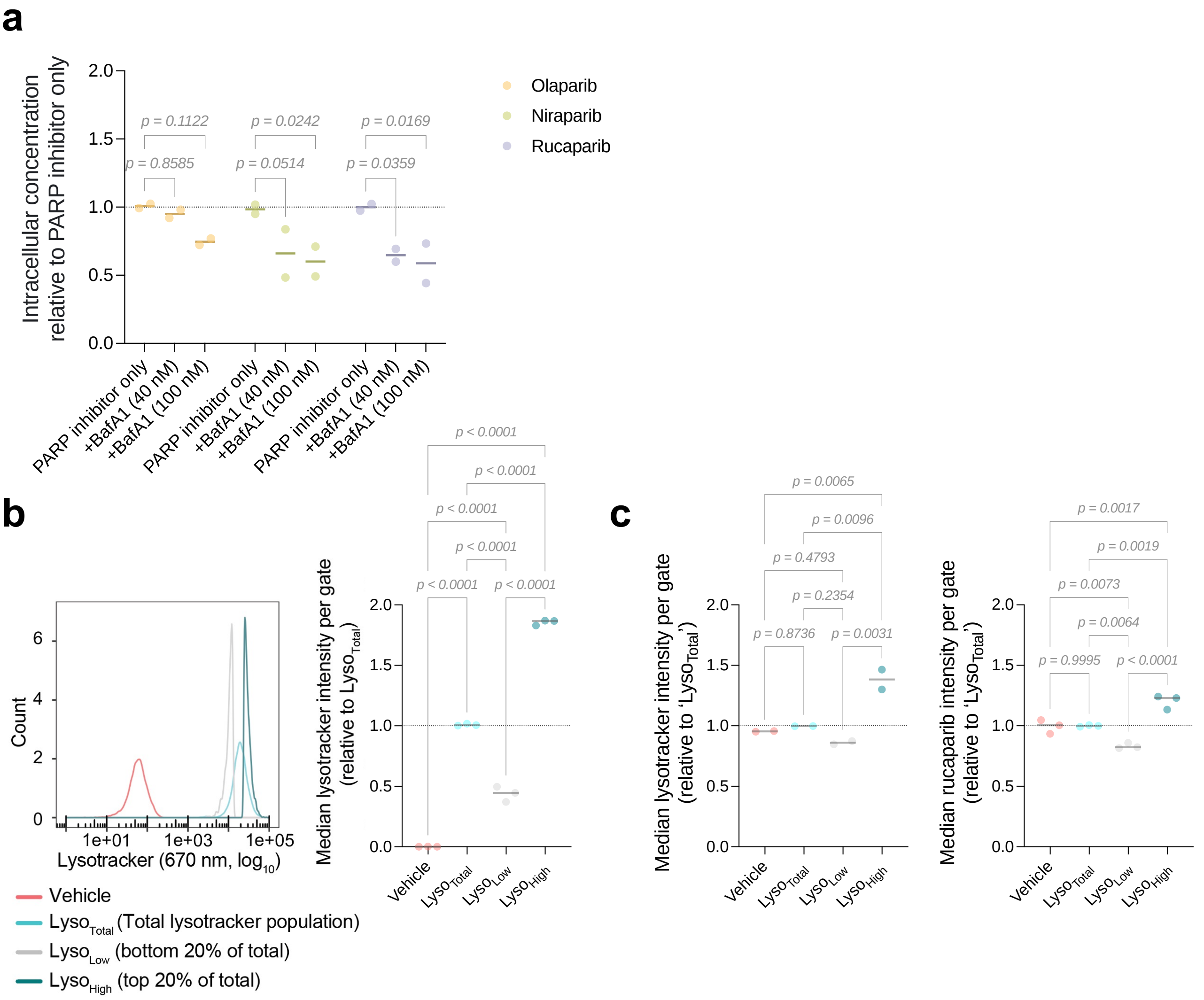

Supplementary Figure 10

Impact of lysosomal accumulation on intracellular PARP inhibitor concentrations (associated with main Figure 6)

A) Intracellular concentration of olaparib, niraparib and rucaparib in OVCAR4 cells with or without treatment with bafilomycin (BafA1) to alkalinise lysosomes. Data derived from 2 biological replicates, and plotted relative to PARP inhibitor only control, within replicate. Statistical significance assessed by 2-way ANOVA with Dunnet's correction for multiple comparisons.

B) $Lyso_{High}$ and $Lyso_{Low}$ PEO1 cells (top and bottom 20% of lysotracker-treated population respectively) were FACS-sorted with total lysotracker and vehicle-only controls after a 30 min treatment. Sorted cells were re-cultured overnight in drug-free media.

C) Intracellular rucaparib and lysotracker levels were assessed by incubating cells with drug or lysotracker for 1 hour prior to FACS analysis (n = 2 biological replicates for lysotracker). For B and C, unless otherwise stated, data depicts n = 3 biological replicates. One-way ANOVA and Tukey's multiple comparison test were applied to evaluate statistical significance.

Source data are provided as a Source Data file.

TFEB antibody

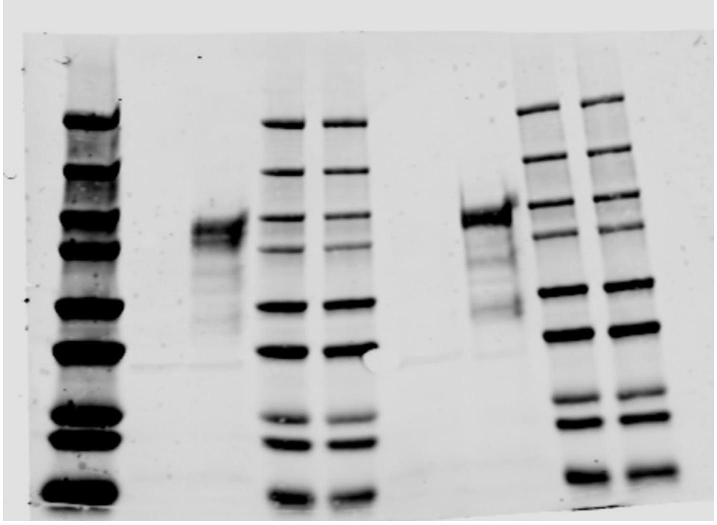

B-actin antibody

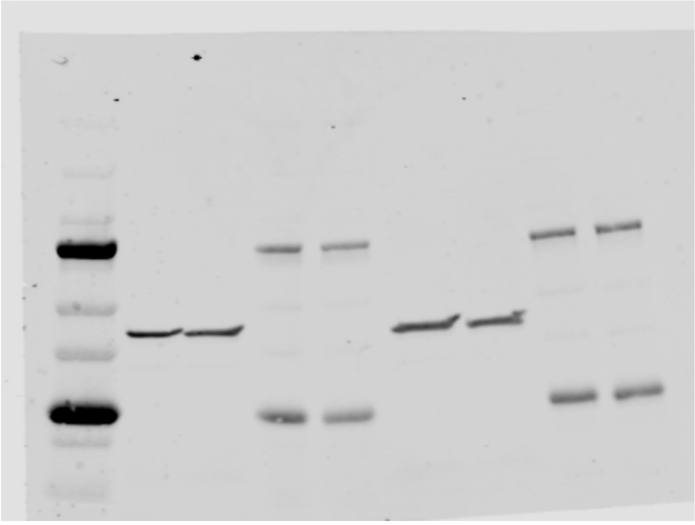

Lane 1 Ladder Precision Plus Protein Dual Color Standards #1610374
Lane 2 Untransfected PEO1 (not boiled)
Lane 3 TFEB-OE PEO1 (not boiled)
Lane 4 Ladder
Lane 5 Ladder
Lane 6 Untransfected PEO1 (boiled)
Lane 7 TFEB-OE PEO1 (boiled)
Lane 8 Ladder
Lane 9 Ladder
