# Supplementary material for: Multimodal imaging reveals a lysosomal drug reservoir that drives heterogeneous distribution of PARP inhibitors
Source: Nat Commun. 2026 Mar 17;17:4086. doi: 10.1038/s41467-026-70558-1 (PMC13144616; doi:10.1038/s41467-026-70558-1)
Supplement: Supplementary file 2 — Description of Additional Supplementary Files [file 41467_2026_70558_MOESM2_ESM.pdf]

Title: Supplementary Data 1
Description: Metadata for tumour samples and corresponding ex vivo slices, including patient and slice identifier, FIGO stage, tumour site and drug treatment.

Title: Supplementary Data 2
Description: Comparative physicochemical characteristics of PARP inhibitors.

Title: Supplementary Data 3
Description: GSEA (Reactome) pathway enrichment analysis comparing high- and low-drug regions of interest across niraparib- and rucaparib-treated PDEs

Title: Supplementary Data 4
Description: Genes whose expression is significantly associated with continuous rucaparib or niraparib concentrations, identified using linear mixed-effects models.

Title: Supplementary Data 5
Description: Gene Ontology Biological Process enrichment analysis of genes identified by linear mixed-effects modelling as significantly associated with continuous niraparib or rucaparib concentrations.

Title: Supplementary Data 6
Description: Gene Ontology Cellular Component enrichment analysis for genes identified by linear mixed-effects modelling as significantly associated with both niraparib and rucaparib concentrations.

Title: Supplementary Data 7
Description: Proteomics analysis of RucHigh versus RucLow PEO1 cell populations.

Title: Supplementary Data 8
Description: Gene Ontology enrichment analysis of proteins significantly enriched in RucHigh versus RucLow PEO1 cells.
