# Supplementary material for: Multimodal imaging reveals a lysosomal drug reservoir that drives heterogeneous distribution of PARP inhibitors
Source: Nat Commun. 2026 Mar 17;17:4086. doi: 10.1038/s41467-026-70558-1 (PMC13144616; doi:10.1038/s41467-026-70558-1)
Supplement: Supplementary file 4 — Reporting Summary [file 41467_2026_70558_MOESM4_ESM.pdf]

nature portfolio

Corresponding author(s): Louise Fets and Zoe Hall

Last updated by author(s): 20 Feb 2026

Reporting Summary

Nature Portfolio wishes to improve the reproducibility of the work that we publish. This form provides structure for consistency and transparency in reporting. For further information on Nature Portfolio policies, see our Editorial Policies and the Editorial Policy Checklist.

Statistics

For all statistical analyses, confirm that the following items are present in the figure legend, table legend, main text, or Methods section.

n/a	Confirmed	
[ ]	[x]	The exact sample size (*n*) for each experimental group/condition, given as a discrete number and unit of measurement
[ ]	[x]	A statement on whether measurements were taken from distinct samples or whether the same sample was measured repeatedly
[ ]	[x]	The statistical test(s) used AND whether they are one- or two-sided<br>*Only common tests should be described solely by name; describe more complex techniques in the Methods section.*
[x]	[ ]	A description of all covariates tested
[ ]	[x]	A description of any assumptions or corrections, such as tests of normality and adjustment for multiple comparisons
[ ]	[x]	A full description of the statistical parameters including central tendency (e.g. means) or other basic estimates (e.g. regression coefficient) AND variation (e.g. standard deviation) or associated estimates of uncertainty (e.g. confidence intervals)
[ ]	[x]	For null hypothesis testing, the test statistic (e.g. *F*, *t*, *r*) with confidence intervals, effect sizes, degrees of freedom and *P* value noted<br>*Give P values as exact values whenever suitable.*
[x]	[ ]	For Bayesian analysis, information on the choice of priors and Markov chain Monte Carlo settings
[x]	[ ]	For hierarchical and complex designs, identification of the appropriate level for tests and full reporting of outcomes
[ ]	[x]	Estimates of effect sizes (e.g. Cohen's *d*, Pearson's *r*), indicating how they were calculated

Our web collection on statistics for biologists contains articles on many of the points above.

Software and code

Policy information about availability of computer code

Data collection	MALDI-Mass Spectrometry Imaging: SMALDIControl (Version 1.6) and ThermoQ Exactive Plus Tune (Version 2.9 SP3 Build 2948)<br>DESI-Mass Spectrometry imaging: HDImaging (Version 1.4) and MassLynx (Version 4.1)<br>IHC: Zeiss microscopy Zen (Version 3.1)<br>Spatial Transcriptomics: GeoMx DSP Analysis Suite (Version 3.1.2.12)<br>Operetta: Harmony (Version 4.9)<br>FACS: BD FACS Diva (Version 9.4)<br>LC-MS: MassLynx (Version 4.2)<br>Proteomics: (Thermo Scientific Excalibur v4.5.474.0)<br>Incucyte S3 (Version 2022B Rev3)<br>SRB: SoftMax Pro (Version 7.3)
Data analysis	MALDI-Mass Spectrometry Imaging: RAW2IMZML (Version 1.8r3), MSiReader (Version 1.02)<br>DESI-Mass Spectrometry Imaging: HDImaging (Version 1.4)<br>IHC: Qupath (Version 0.5.0)<br>Spatial Transcriptomics: GeoMx DSP Analysis Suite (Version 3.1.2.12)<br>Operetta: Harmony (Version 4.9), CellProfiler (Version 4.2.8) and ImageJ2 (Version 2.16.0/1.54p)<br>FACS: FlowJo (Version 10.10.0)<br>LC-MS: (Skyline(64-bit) 24.1.0.414)<br>Proteomics: Spectronaut (Version 19.0.240606.62635) and Perseus (Version 1.6.15.0)

Graph plotting and statistical analysis: Graphpad Prism (Version 10.4.1)
In-house scripting: RStudio (2025.05.0+496), running R version 4.4.1 (2024-06-14)

For manuscripts utilizing custom algorithms or software that are central to the research but not yet described in published literature, software must be made available to editors and reviewers. We strongly encourage code deposition in a community repository (e.g. GitHub). See the Nature Portfolio guidelines for submitting code & software for further information.

Data

Policy information about availability of data

All manuscripts must include a data availability statement. This statement should provide the following information, where applicable:

- Accession codes, unique identifiers, or web links for publicly available datasets
- A description of any restrictions on data availability
- For clinical datasets or third party data, please ensure that the statement adheres to our policy

Mass spectrometry proteomics data have been deposited to the ProteomeXchange Consortium via the PRIDE partner repository with the dataset identifier PXD057265 [https://www.ebi.ac.uk/pride/archive?keyword=pxd057265]. Spatial transcriptomics data are available from GEO under accession code GSE281519 [https://www.ncbi.nlm.nih.gov/geo/query/acc.cgi?acc=GSE281519]. To accommodate the high complexity and volume of mass spectrometry imaging data, we have provided processed analysis outputs and representative ion maps within the Source Data files and main text to ensure all findings are fully verifiable. The remaining data are available within the article, supplementary information, or source data file. Source data are provided with this paper.

Research involving human participants, their data, or biological material

Policy information about studies with human participants or human data. See also policy information about sex, gender (identity/presentation), and sexual orientation and race, ethnicity and racism.

Reporting on sex and gender	This study focused on ovarian cancer, a disease suffered only by genetically female patients. As such, all cell lines and patient samples were genetically female in origin.
Reporting on race, ethnicity, or other socially relevant groupings	Data on race, ethnicity of other social groupings of patients who donated samples were not available to us. Importantly, this was a small feasibility study, which did not have sufficient statistical power for this level of stratification.
Population characteristics	as above.
Recruitment	Patients undergoing surgery for advanced High Grade Serous Ovarian Carcinoma at Hammersmith Hospital, London, UK, a tertiary gynecological cancer centre certified by the European Society of Gynaecological Oncology (ESGO) as a centre of excellence for ovarian cancer surgery.
Ethics oversight	The human samples from this research project were banked by the Imperial College Healthcare Tissue Bank (ICHTB). ICHTB us supported by the National Institute for Health Research (NIHR) Biomedical Research Centre based at Imperial College Healthcare NHS Trust and Imperial College London. ICHTB is approved by Wales REC3 to release human material for research (22/WA/2836). All patients gave written consent. The procedures involving human participants were done in accordance with the ethical standards of the institutional and/or national research committee and with the principles of the 1964 Declaration of Helsinki and its later amendments or comparable ethical standards.

Note that full information on the approval of the study protocol must also be provided in the manuscript.

Field-specific reporting

Please select the one below that is the best fit for your research. If you are not sure, read the appropriate sections before making your selection.

[x] Life sciences [ ] Behavioural & social sciences [ ] Ecological, evolutionary & environmental sciences

For a reference copy of the document with all sections, see nature.com/documents/nr-reporting-summary-flat.pdf

Life sciences study design

All studies must disclose on these points even when the disclosure is negative.

Sample size	In the case of spatial transcriptomics analyses, GeoMx DSP was performed on 5 high-drug and 5-low-drug regions of interest from each explant, which captured the variability within each sample. For LC-MS, a minimum of two biological replicates, each with 4 technical replicates, were analysed. For imaging and FACS based experiments, 3 biological replicates were obtained on different days, each with a minimum of 3 technical replicates (derived from separate wells). Specific details of sample sizes for every experiment are provided in figure legends.
Data exclusions	In LC-MS experiments, ROUT outlier detection test (Q = 5%) was applied
Replication	All attempts at replication were successful.
Randomization	Sample sizes were small enough in this study that randomisation was not applicable.
Blinding	Blinding was not used.

Reporting for specific materials, systems and methods

We require information from authors about some types of materials, experimental systems and methods used in many studies. Here, indicate whether each material, system or method listed is relevant to your study. If you are not sure if a list item applies to your research, read the appropriate section before selecting a response.

Materials & experimental systems

n/a	Involved in the study
☐	☒ Antibodies
☐	☒ Eukaryotic cell lines
☒	☐ Palaeontology and archaeology
☒	☐ Animals and other organisms
☒	☐ Clinical data
☒	☐ Dual use research of concern
☒	☐ Plants

Methods

n/a	Involved in the study
☒	☐ ChIP-seq
☐	☒ Flow cytometry
☒	☐ MRI-based neuroimaging

Antibodies

Antibodies used

Immunohistochemistry primary antibodies:
Wilms Tumour Protein (WT1) Abcam, ab89901 CANR9(IHC)-56-2 Rabbit 1:500
Pax8 Abcam, ab189249 EPR13511 Rabbit 1:1000
Phospho histone H2A.X (Ser139) CST, 2577 Rabbit 1:400
Cleaved Caspase-3 (Asp175) CST, 9664 5A1E Rabbit 1:100

Spatial Transcriptomics antibodies:
Pan cytokeratin (PanCK) Novus, NBP2-33200 AE1+AE3 532 80 µg/ml
CD45 Novus, NBP2-34528 2B11+PD7/26 594 320 µg/ml
Alpha-smooth muscle actin (α-SMA) Abcam, ab267537 SP171 647 0.5 µg/ml

Immunocytochemistry antibodies:
Phospho histone H2A.X (Ser139) CST 2577 Rabbit 1:1000
α-Tubulin Sigma T9026 DM1A Mouse 1:1000
Alexa Fluor 488 Rabbit A21206 1:1000
Alexa Fluor 555 Rabbit A31572 1:1000
Alexa Fluor 568 Mouse A10037 1:1000
Alexa Fluor 647 Mouse A31571 1:1000

Western blotting antibodies:
TFEB (D207D), CST, 37785, Rabbit 1:1000
β-actin (AC74), SIGMA, A5316, Mouse 1:1000

Validation

Validation of antibodies was performed by manufacturers, details can be found using the catalogue numbers listed above.

Eukaryotic cell lines

Policy information about cell lines and Sex and Gender in Research

Cell line source(s)

PEO1 and 4 were a gift from Simon Langdon. All other cell lines were obtained from ATCC.

Authentication

All cell lines were recently authenicated using STR profiling from either ATCC Cell Line Authentication or Eurofins

Mycoplasma contamination

All cell lines were tested mycoplasma-free with an in-house testing kit on a monthly basis.

Commonly misidentified lines
(See ICLAC register)

No commonly misidentified cell lines were used.

Plants

Seed stocks | n/a

Novel plant genotypes | n/a

Authentication | n/a

Flow Cytometry

Plots

Confirm that:

- [x] The axis labels state the marker and fluorochrome used (e.g. CD4-FITC).
- [x] The axis scales are clearly visible. Include numbers along axes only for bottom left plot of group (a 'group' is an analysis of identical markers).
- [ ] All plots are contour plots with outliers or pseudocolor plots.
- [ ] A numerical value for number of cells or percentage (with statistics) is provided.

Methodology

Sample preparation	Cells were treated with Rucaparib at IC50 concentration or vehicle only for 1, 2 or 24 hours. In some experiments cells were optionally treated with Lysotracker Deep Red at 50 nM and Rucaparib for 1 hour. Following the incubation period, cells were rinsed with PBS, harvested using TrypLE express and resuspended at a concentration of 5–10 million cells/mL in ice-cold PBS or phenol red-free RPMI supplemented with 2% FCS, and optionally 100 U/mL penicillin, 100 µg/mL streptomycin. Cell suspensions were then transferred to polystyrene test tubes through a 35 µm cell strainer cap and kept on ice until sorting. Rucaparib was excited by the 355nm laser and emission was collected using a 450/50 nm bandpass filter. Lysotracker was excited by the 640 nm laser and emission was collected using a 670/30 nm bandpass filter.
Instrument	FACSAria Fusion cell sorter (BD Biosciences)
Software	BD FACS Diva (Version 9.4) and FlowJo (Version 10.10.0)
Cell population abundance	Post-sort cell fractions were collected based on Rucaparib fluorescence intensity, with the top and bottom 20% of the Rucaparib-positive distribution sorted from the total live cell pool. Each fraction yielded approximately 1 million cells. Unlike conventional fluorophore-conjugated antibodies, Rucaparib is a small-molecule compound whose fluorescent signal diminishes over time due to intracellular processing and washout, making immediate post-sort purity assessment by re-analysis unreliable. While samples were not re-run immediately post-sort, functional separation of the populations was confirmed: after 3 weeks in culture and re-treatment with Rucaparib, cells originally sorted as "high" retained significantly greater drug uptake than those from the "low" (bottom 20%) population, indicating successful enrichment at the time of sorting.
Gating strategy	The initial gating strategy included standard forward scatter (FSC-A) versus side scatter (SSC-A) to exclude debris based on size and granularity. This was followed by gating on FSC-A versus FSC-H to select singlet events and eliminate doublets or aggregates. For Rucaparib signal detection, cells were excited using the 355 nm laser and emission collected using a 450/50 nm bandpass filter. Positive and negative boundaries for Rucaparib fluorescence were defined using vehicle-treated controls as negative references. The top and bottom 20% of the Rucaparib-positive distribution were then selected for sorting, as depicted in Figure 4a.

- [x] Tick this box to confirm that a figure exemplifying the gating strategy is provided in the Supplementary Information.
